# Disorder in Order-Related Membrane Biophysical Parameters: An In-Depth Analysis of Di-4-ANEPPDHQ Generalized Polarization

**DOI:** 10.1021/acs.jpclett.6c00048

**Published:** 2026-04-10

**Authors:** Rosemary Chandrakanthi Kothalawala, Csenge Makay, Lajos Szente, Zoltan Varga, Gyorgy Panyi, Peter Nagy, Florina Zakany, Tamas Kovacs

**Affiliations:** † Department of Biophysics and Cell Biology, Faculty of Medicine, University of Debrecen and MTA Centre of Excellence, Hungarian Academy of Sciences, Egyetem tér 1, Debrecen H-4032, Hungary; ‡ 368105 CycloLab Cyclodextrin R&D Laboratory Ltd., Illatos u. 7., Budapest H-1097, Hungary

## Abstract

Molecular order-related bulk membrane properties that substantially modulate protein functions can be examined with environment-sensitive probes, such as the prototypical and most widely applied solvatochromic Laurdan, whose spectral parameters change depending on the local hydrophobicity. Di-4-ANEPPDHQ is a widely accepted Laurdan alternative with more favorable spectral properties suitable for standard imaging, and information provided by the two fluorophores is generally considered equivalent. In our study, using fluorescence-based experimental approaches, we demonstrate that different sterols distinctly alter di-4-ANEPPDHQ spectral properties, and these changes do not correlate with those observed with Laurdan. Our molecular dynamics simulations reveal that this may be caused by their distinct depth localization in bilayers since the sensor moiety of di-4-ANEPPDHQ is localized in the vicinity of the membrane–water interface as opposed to that of Laurdan lying near the hydrophobic core. Therefore, di-4-ANEPPDHQ can be considered as a complementary tool rather than an equivalent substitute of Laurdan.

## Graphical abstract

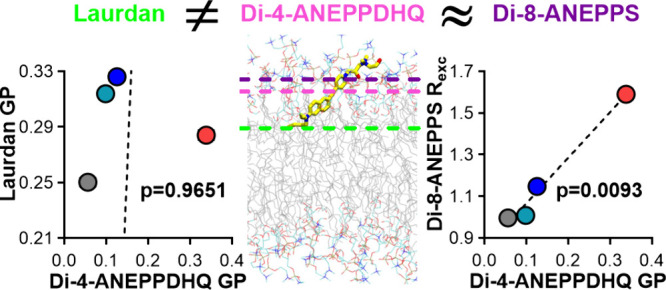

The bulk structural organization is an essential, intrinsic, and dynamic property of all biological membranes, which plays a fundamental active role in the modulation of membrane protein activity and, therefore, a wide variety of cellular functions.^1,2^ However, examination of such membrane features is often neglected in the new era of cutting-edge structural techniques, such as X-ray crystallography or cryoelectron microscopy, in studies of protein structures and direct lipid–protein interactions, which is also partially attributable to the scarcity of experimental methods suitable for the investigation of membrane organization in living cells. For this purpose, environment-sensitive fluorophores can be utilized in intact cells, which change their fluorescence properties in response to alterations in the local microenvironment. These dyes are routinely classified based on the mechanism of sensitivity with the largest families comprising viscosity-sensitive fluorescent probes, polarity-sensitive solvatochromic dyes, and voltage-sensitive electrochromic dyes.^3,4^ These major groups of probes characterize three different aspects of molecular organization in the membrane, i.e., membrane fluidity, hydration, and dipole potential, respectively. From among these order-related parameters, fluidity is defined as the rotational freedom of molecules, hydration is defined as the extent of penetration of water into the bilayer, whereas dipole potential represents a large positive intramembrane potential arising from the nonrandom orientation of molecular dipoles at the membrane–water interface.^5–9^ Since these properties are essentially linked to each other, they are often collectively termed “membrane order” or “lipid order” and results obtained with the different environment-sensitive dyes are considered equivalent in spite of sporadic results demonstrating incongruent changes in the fluorescence properties of the dyes in response to alterations in membrane lipid composition.^10–12^ In accordance with the latter, in our previous study with fluorescence-based experimental approaches, we demonstrated dissimilar changes in the fluorescence properties of four commonly applied environment-sensitive dyes elicited by different sterols, and using molecular dynamics simulations, we showed that the distinct sensitivity of the dyes is caused by the incorporation of their sensor moieties at different bilayer depths.^13^

Solvatochromic dyes are frequently utilized to describe molecular order in membranes, and Laurdan (6-dodecanoyl-*N*,*N*-dimethyl-2-naphthylamine) is the founding prototypical member of the family since its emission spectrum is altered as a function of the local hydrophobicity.^14,15^ Namely, its emission spectrum is red-shifted in a hydrophilic environment due to solvent relaxation, i.e., a rapid reorganization of water molecules in the vicinity thereby decreasing the energy of the excited state. The generalized polarization of the probe quantifying the extent of this spectral shift thus negatively correlates with the degree of water penetration into the membrane, and its quantification is widely utilized in living cells to report on membrane structure.^10,11,16–20^ However, its applicability is limited by its unfavorable spectral properties; in other words, its excitation spectrum falls in the near-UV range. Therefore, the generalized polarization of Laurdan is typically determined with spectrofluorometry, which lacks the ability to provide information about individual cells^10,11,17^ or two-photon microscopy, which is not widely available.^16,18,20,21^

Di-4-ANEPPDHQ was introduced as an environment-sensitive probe having more favorable, red-shifted spectra suitable for imaging with conventional microscope setups.^22,23^ Upon examination in combination with Laurdan, the two fluorophores gave similar results, and consequently, di-4-ANEPPDHQ became accepted as a Laurdan alternative to characterize membrane structure.^19,24–30^ However, sporadic results showed that the information provided by the two dyes is not necessarily equivalent, and therefore, di-4-ANEPPDHQ cannot be considered as a full-fledged alternative of Laurdan.^31,32^ In our study, using fluorescence-based approaches, we also provide evidence that the two dyes may exhibit incongruent responses due to, as revealed by our molecular dynamics simulations, distinct depth localization in bilayers.

The structural organization and therefore the biophysical properties of bilayers substantially depend on the quality and quantity of membrane lipids. Membrane cholesterol is one of the major determinants of membrane structure since its concentration negatively correlates with membrane fluidity^12,17,33,34^ and hydration^12,17–19,25,35^ through inducing the stretching of acyl chains of phospholipids, altering penetration and ordering of membrane-associated water molecules. Due to these same membrane-ordering properties, the cholesterol concentration positively correlates with the dipole potential.^10,12,36–41^ Other sterols also modify membrane organization but often in a distinct manner. For example, 7-dehydrocholesterol similarly reduces fluidity and hydration^12,42^ by inducing smaller increases in the dipole potential.^12,37,43^ Conversely, 6-ketocholestanol largely increases the dipole potential^10,12,44–46^ without causing notable changes in fluidity or hydration.^10,12^ The altered effects on the membrane structure of these sterols may be related to distinct magnitudes of their dipole moment, tilt angles in the bilayer, different depth localization in the membrane, and effects on penetration and ordering of membrane-associated water molecules.

Due to the uncertainty regarding which membrane biophysical property di-4-ANEPPDHQ reports, the aim of this work is to correlate its response to cholesterol analogues with that of Laurdan (a probe of membrane hydration) and di-8-ANEPPS (a reporter of dipole potential). Changing the levels of cholesterol, 7-dehydrocholesterol, and 6-ketocholestanol induces distinct alterations in membrane structure, which enables studying the sensitivity of environment-sensitive fluorophores to different aspects of membrane organization as performed in our previous study.^13^ These sterols can be experimentally incorporated into the membranes by using their inclusion complexes with randomly methylated β-cyclodextrin (MβCD). In native empty form, MβCD depletes membrane cholesterol, whereas when precomplexed, it can efficiently load bilayers with sterols.^5,12,43,47,48^ However, precomplexation results in a mixture of empty and sterol-loaded MβCD, and as a result, two competing processes occur during treatment of cells, that is, cholesterol depletion and sterol loading. Therefore, MβCD-treated samples should serve as controls in these experiments.^12,43,48^

In accordance, to examine effects on di-4-ANEPPDHQ generalized polarization, we treated CHO cells with MβCD precomplexed with cholesterol (CHOL), 7-dehydrocholesterol (7DHC), and 6-ketocholestanol (6KC) and empty MβCD as a control. After treatment and thorough washing, we labeled the cells with di-4-ANEPPDHQ and followed changes in the emission spectrum of the dye using spectrofluorometry. In these experiments, we observed blue-shifts in the spectrum in response to all three sterols when compared to the control, implying a more hydrophobic microenvironment, that is, less membrane hydration (Figure 1A). However, the degree of shifts was notably different among the sterols. We determined the extent of these shifts by calculating the generalized polarization, and significant increases were found when comparing each sterol to the control and between the sterols, as well, with values for the different treatments decreasing in the following order: 6KC ≫ CHOL > 7DHC > MβCD (Figure 1B). Since spectrofluorometry does not provide information about individual cells and the main advantage of di-4-ANEPPDHQ over Laurdan is its suitability for conventional imaging, we performed these measurements with confocal microscopy, as well. During quantitative image analysis, we identified individual cells and calculated the median value of generalized polarization exclusively in the plasma membranes of individual cells (Figure 1C). We found strongly concordant results with those seen using spectrofluorometry with di-4-ANEPPDHQ generalized polarization values in the following order: 6KC ≫ CHOL > 7DHC > MβCD (Figure 1D).

**Figure 1:**
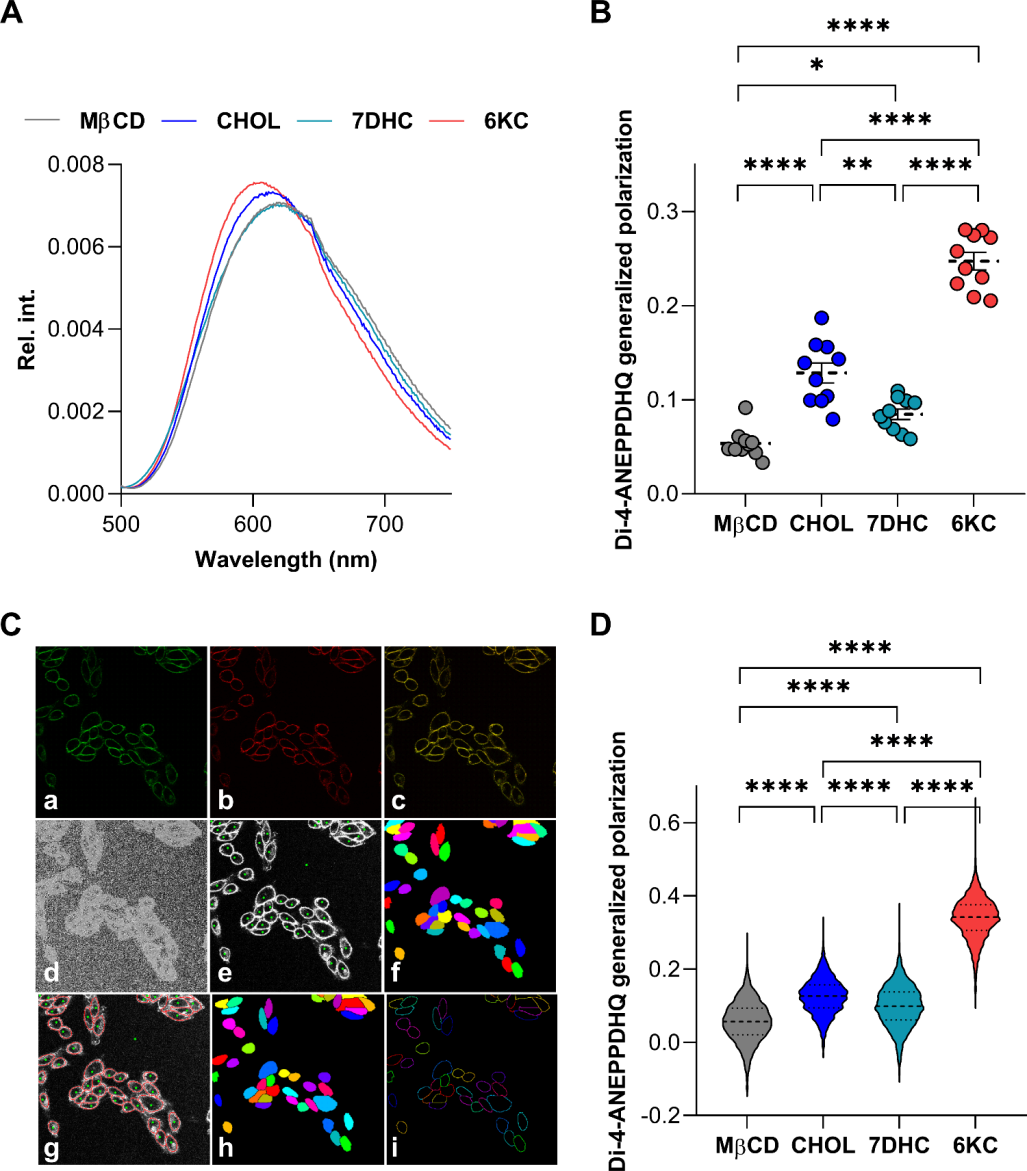
Effects of cyclodextrin-complexed sterols on the generalized polarization of di-4-ANEPPDHQ. (A) Detached CHO cells were treated for 1 h with native MβCD and cholesterol–MβCD (CHOL), 7-dehydrocholesterol–MβCD (7DHC), and 6-ketocholestanol–MβCD (6KC) complexes and stained with di-4-ANEPPDHQ. Emission spectra were acquired after a 488 nm excitation using spectrofluorometry. (B) Individual di-4-ANEPPDHQ generalized polarization values were subsequently obtained from the spectra in *n* = 10 independent samples containing approximately 100 000 cells, and their average values (±SEM) are plotted in the figure. (C) CHO cells grown on an eight-well chambered coverglass were treated and stained as described above. Representative confocal microscopic images taken at the midplane of cells show di-4-ANEPPDHQ intensities measured in the (a) blue and (b) red wavelength ranges, and (c) their overlay. During quantitative image analysis, (d) the generalized polarization was calculated on a pixel-by-pixel basis and (e) a custom-written manually seeded watershed algorithm was applied to identify (f) the cells and (g) the pixels corresponding to the plasma membrane of cells. (h) This was followed by the identification of individual cells and, consequently, (i) plasma membrane pixels of individual cells. (D) Violin plots were generated from median di-4-ANEPPDHQ generalized polarization values calculated exclusively from pixels corresponding to the plasma membrane in *n* = 3500–4000 individual cells per treatment condition obtained from five independent experiments, which also display median values with quartiles. Asterisks indicate significant differences between the samples (*p < 0.05, **p < 0.01, and ****p < 0.0001; ANOVA followed by Tukey’s HSD test).

In the next step, we compared our results with those obtained in our previous study after identical treatments and experimental conditions with solvatochromic Laurdan and electrochromic di-8-ANEPPS.^13^ We performed Deming regression analysis between di-4-ANEPPDHQ generalized polarization and Laurdan generalized polarization or di-8-ANEPPS excitation ratio values obtained after MβCD, CHOL, DHC, and 6KC. No statistically significant correlation was found between generalized polarization values of di-4-ANEPPDHQ and Laurdan (Figure 2A). On the contrary, very strong, statistically significant positive correlation was seen between di-4-ANEPPDHQ generalized polarization and the di-8-ANEPPS excitation ratio (Figure 2B). This supports the hypothesis that in contrast to the widespread belief that both di-4-ANEPPDHQ and Laurdan share the same solvatochromic sensing mechanism, di-4-ANEPPDHQ cannot be considered a perfect alternative of Laurdan and suggests that the information provided by the dye in fact resembles much more closely that gained with di-8-ANEPPS, a probe with a presumably distinct sensing mechanism, electrochromism.

**Figure 2:**
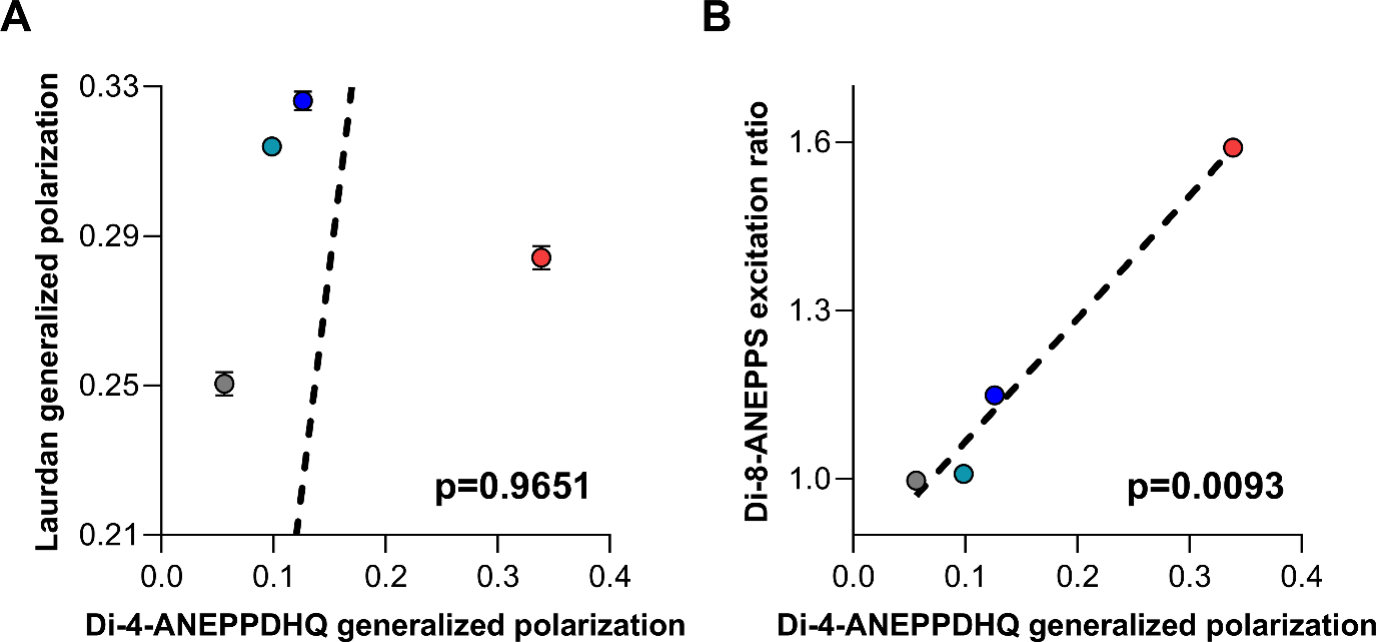
Comparison of the effects of cyclodextrin-complexed sterols on the generalized polarization of di-4-ANEPPDHQ, the generalized polarization of Laurdan, and the excitation ratio of di-8-ANEPPS. (A) Laurdan generalized polarization plotted as a function of di-4-ANEPPDHQ generalized polarization. (B) Di-8-ANEPPS excitation ratio plotted as a function of di-4-ANEPPDHQ generalized polarization. Average values (±SEM) of the Laurdan generalized polarization and di-8-ANEPPS excitation ratio, determined in our previous study^13^ under identical experimental conditions, are compared with di-4-ANEPPDHQ generalized polarization values measured in the present study in cells treated with native MβCD (gray) and cholesterol–MβCD (CHOL, dark blue), 7-dehydrocholesterol–MβCD (7DHC, cyan), and 6-ketocholestanol–MβCD (6KC, red) complexes. The *p* values determined with Deming regression analysis are shown in the panels, which revealed a significant correlation only between the di-4-ANEPPDHQ generalized polarization and di-8-ANEPPS excitation ratio.

To investigate why the results obtained with di-4-ANEPPDHQ correlate more strongly with those of di-8-ANEPPS than with those of Laurdan, we examined the membrane localization of di-4-ANEPPDHQ using MD simulations following the positions of its heavy atoms (Figure 3A). We embedded the dye into a 1-palmitoyl-2-oleoyl-*sn*-glycero-3-phosphocholine (POPC) lipid bilayer at two different initial depths, model 1 and model 2, with the latter shifted 5 Å toward the bilayer center (Figure 3B). Overall, time-dependent examination of membrane thickness measured between the P atoms in two leaflets, tilt angles of the dye to membrane normal, and positional RMSDs of heavy atoms of the probe suggested that the system gained and maintained stability during the simulations (Figure 3C). We also followed time-dependent changes in the distance of N2 and C23 atoms at the two opposing edges of the chromophore moiety from the P atoms of phospholipids at the proximal interface (Figure 3D). Subsequently, we calculated the average position of the selected atoms in the last 100 ns interval of each simulation (Figure 3E). We found that the chromophore moiety was localized, on average, 4.4 Å from the membrane–water interface, which was much closer to the average depth localization of the sensor moiety of di-8-ANEPPS than to Laurdan, the two latter values determined in our previous study with identical simulation methods and conditions (3.7 and 10.6 Å, respectively).^13^ Notably, the two simulations, although the fluorophore was positioned at different initial depths, yet yielded similar results since, after approximately 80 ns, localization of the dye in model 2 converged to that in model 1, and the trajectories became essentially indistinguishable for the remainder of the simulation time, which further supports our conclusions. Altogether, our MD simulations provided an explanation for our experimental results, showing that data gained with di-4-ANEPPDHQ are much more similar to those of di-8-ANEPPS rather than Laurdan. That is, the sensor moiety of di-4-ANEPPDHQ lies much closer to the membrane–water interface than that of Laurdan.

**Figure 3:**
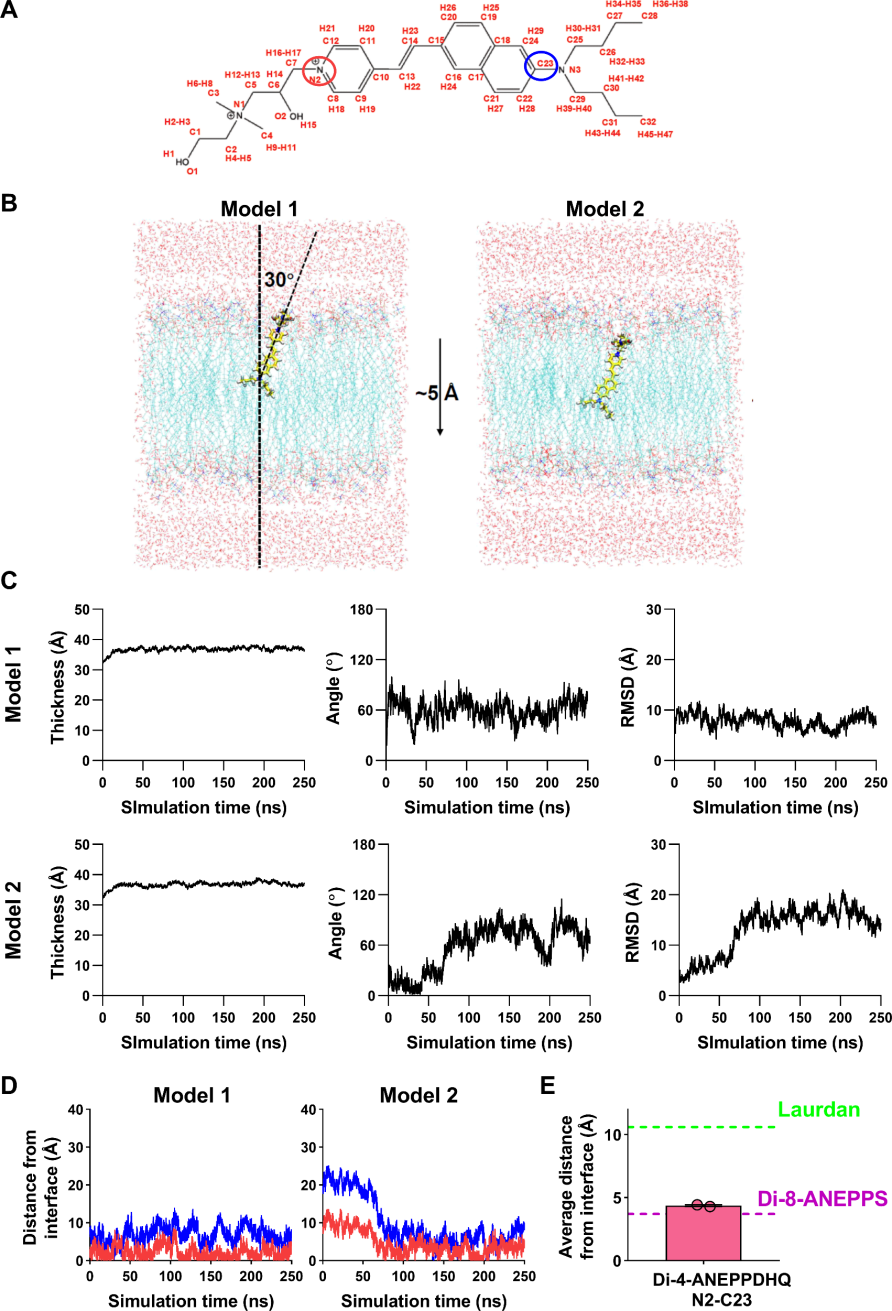
Membrane localization of the chromophore moiety of di-4-ANEPPDHQ. (A) Schematic structure of di-4-ANEPPDHQ displayed with the name of each atom labeled in red. The atoms selected for further analysis are circled with the red and blue circles showing the boundaries of the chromophore groups. (B) Di-4-ANEPPDHQ was embedded into a pre-equilibrated lipid bilayer composed of 1-palmitoyl-2-oleoyl-*sn*-glycero-3-phosphocholine (POPC) with the initial orientation of the fluorophore having a 30° angle to the membrane normal with the tilt angle to membrane normal measured based on atoms N2 and N3, and the dye localized at two different initial depths shifted by 5 Å (model 1 and model 2). The tilt angle is labeled in model 1. (C) Membrane thickness values measured between the P atoms in the top and bottom layers, tilt angles to the membrane normal, and positional RMSDs of the heavy atoms of the dye were plotted as a function of simulation time during the course of the MD simulation and demonstrate the overall stability of the system. (D) Distances of the N2 (red) and C23 (blue) atoms at the two opposing edges of the chromophore moiety of di-4-ANEPPDHQ were calculated to the membrane surface defined as the position of the P atoms in phospholipids and plotted as a function of simulation time. (E) Mean positions of the selected atom pairs were calculated in the last 100 ns intervals of simulations and compared with those of atoms at the two opposing edges of the sensor moieties of Laurdan and di-8-ANEPPS determined in our previous study.^13^

Both experimental and MD simulation data obtained in this study suggest that di-4-ANEPPDHQ is not an ideal alternative for Laurdan. In fact, the information provided by the two dyes is complementary rather than equivalent. Di-4-ANEPPDHQ was originally synthesized as a fluorophore having the same chromophore as potentiometric di-8-ANEPPS and the quaternary ammonium headgroup (DHQ) of the dienylstyryl-pyridinium dye RH795 with good water solubility and photostability, a low degree of internalization, and excitation falling in the range of standard microscope setups.^23^ Its emission spectrum was demonstrated to undergo a notable blue-shift in cholesterol-enriched liquid-ordered model bilayers and lipid rafts of living cells, as well, making it a “phase-sensitive” probe, which was suggested to include both solvatochromic and electric field-dependent, electrochromic components. However, the former was deemed to provide a larger contribution to the observed spectral response.^22^ Based on this assumption, di-4-ANEPPDHQ became widely known as a polarity-sensitive solvatochromic probe. In accordance, most studies simultaneously examining Laurdan, the prototypical solvatochromic dye, and di-4-ANEPPDHQ yielded similar results, and therefore, di-4-ANEPPDHQ became a generally accepted Laurdan alternative and information obtained with the two fluorophores was considered equivalent.^19,24–28^ However, a thorough analysis of their steady state and time-resolved spectra in different solvents and phase-separated cell-derived giant plasma membrane vesicles suggested that distinct underlying mechanisms lay behind their spectral changes and thus the two fluorophores may report on different physicochemical membrane properties. The authors proposed that while changes in the general polarization of Laurdan are completely consistent with the typical solvent dipolar relaxation process, those of di-4-ANEPPDHQ may involve multiple mechanisms, including an electrochromic response.^32^ Similar measurements performed in nanodiscs and liposomes also argued against the fully solvatochromic mechanism of the dye.^31^

Due to the inconsistent terminology of membrane biophysics in general and the only partially elucidated environment-sensing mechanism of di-4-ANEPPDHQ in particular, the interpretation of results obtained with the dye seems a bit confusing. A large fraction of recent studies attribute changes reported by the dye in simple terms and somewhat ambiguously to “membrane order” ^49–52^ or “lipid packing” ^53–55^ without addressing further details. When specified, “polarity-sensitive” di-4-ANEPPDHQ was most commonly suggested to report on membrane hydration;^29,30^ however, “fluidity” and “rigidity” are also commonly used terms to interpret results gained with the probe.^56–60^ Di-4-ANEPPDHQ was also described recently as a “voltage-sensitive” dye characterizing “membrane fluidity”.^61^ Furthermore, the depth localization of the probe and its relationship with the environmental sensitivity are not discussed in these studies. Based on our results, di-4-ANEPPDHQ reports on the molecular organization of membrane layers in the vicinity of the membrane–water boundary and, therefore, can be considered as a rather superficial probe, just like the structurally related di-8-ANEPPS, and in contrast with Laurdan that is located much closer to the hydrophobic core and thus can be considered as a deep probe. Both experimental evidence and MD simulation evidence presented in the study support the idea that di-4-ANEPPDHQ is not a substitute but rather a complementary tool for Laurdan. Our data may also imply that the classification of environment-sensitive dyes should be based on depth localization rather than the exact and often not fully understood molecular sensing mechanism. Moreover, the membrane structure cannot be thoroughly described without a combination of dyes reporting on distinct layers at different depths.

Furthermore, our results extend previous sporadic studies demonstrating incongruent changes in membrane structure in response to alterations in levels of certain sterols at different depths of bilayers.^10–12^ The three sterols applied in the study, cholesterol, 7-dehydrocholesterol, and 6-ketocholestanol, albeit having subtle differences in their chemical structures, induce distinct alterations in membrane layers at different depths and hence can be used as experimental tools to elucidate modulatory effects exerted by the bulk membrane structure on transmembrane proteins. Alterations in bulk membrane organization were previously shown to affect a variety of proteins, including receptor tyrosine kinases, G protein-coupled receptors, ion channels, and ATP-driven pumps, through hydrophobic mismatch, elastic coupling, or interactions between the dipole potential-associated electric field and charged or polar amino acid residues.^2,5,6,8,9,62,63^ Investigating changes in membrane structure and their roles in modulating protein activities and thus cellular functions may be crucially relevant. Such studies could help explore novel routes of membrane lipid therapy aimed at correcting membrane composition and structure in diseases associated with altered membrane lipid levels, such as metabolic disorders, lysosomal storage diseases, neurodegenerative diseases, and various tumors.^6,64,65^

Limitations of the simulations in the current study include the use of pure POPC bilayers instead of complex, sterol-containing ones, which would better mimic real cellular membranes, to ensure comparability with prior studies examining the localization of environment-sensitive fluorophores.^66–71^ Moreover, Laurdan can assume an “elongated” or an “L-shaped/bent” conformation in the membrane,^72^ and the distribution between the two conformers may also contribute to its environmental sensitivity.^73,74^ Therefore, it cannot be considered a simple stick standing as a purely solvatochromic reporter in the membrane; it may rather have a phase-dependent molecular rotor behavior, as well.^75–77^ However, as a simplification in our prior comparative analysis, we modeled only the elongated form that, as recent studies demonstrated, may predominate in biological bilayers.^78^ In the experimental part of our work, we investigated the effects of sterols only on the generalized polarization of di-4-ANEPPDHQ. While this parameter is most commonly examined when the fluorophore is used,^22,24,29,32,61,79^ other properties could also be measured, such as lifetime,^25,80–82^ anisotropy,^83^ or time-resolved emission and relaxation dynamics.^31,32^ Furthermore, while the utilized sterols carry both experimental and biological relevance, no systematic analysis of various lipid classes was performed in our measurements. Nevertheless, our strongly congruent experimental and computational results strongly support the relevance of the functional distinction between di-4-ANEPPDHQ and Laurdan.

In summary, using spectrofluorometry and confocal microscopy, we demonstrate here that different but structurally similar sterols induce distinct changes in the generalized polarization of the supposedly solvatochromic environment-sensitive di-4-ANEPPDHQ fluorophore, a proposed functional analogue of Laurdan, the founding member of solvatochromic probes. Somewhat surprisingly, these alterations exhibited no statistically significant correlation with Laurdan generalized polarization values but, contrastingly, showed excellent and significant positive correlations with changes in the dipole potential-dependent excitation ratios of the electrochromic di-8-ANEPPS fluorophore. Our MD simulations demonstrated that this may be related to the depth localizations of the fluorophores since the sensor moiety of di-4-ANEPPDHQ was found in the vicinity of the membrane–water interface, much closer to that of di-8-ANEPPS than that of Laurdan, with the latter being localized much deeper in the hydrophobic core regions. Altogether, our findings imply that the information provided by di-4-ANEPPDHQ may be complementary rather than equivalent to that obtained with the solvatochromic Laurdan and resembles much more closely that obtained with voltage-sensing di-8-ANEPPS. Therefore, conclusions based on the general assumption that di-4-ANEPPDHQ and Laurdan are functionally equivalent should be treated with caution. Furthermore, based on our results, the classification of environment-sensitive fluorophores should include their depth localization in the bilayers rather than focusing solely on the mechanism of sensation, and the membrane biophysical terms should be more unambiguously used when interpreting results gained with these dyes.

## Supplementary Material



## Notes

Experimental section with a detailed description of the utilized cell culture and treatments, examination of spectral changes of di-4-ANEPPDHQ using spectrofluorometry, quantification of the generalized polarization of di-4-ANEPPDHQ using confocal microscopy, molecular dynamics simulations, and statistical analysis (PDF)

## Abbreviations

6KC: 6-ketocholestanol
7DHC: 7-dehydrocholesterol
CD: cyclodextrin
CHOL: cholesterol
di-8-ANEPPS: 4-(2-[6-(dioctylamino)-2-naphthalenyl]­ethenyl)-1-(3-sulfopropyl)­pyridinium inner salt
Laurdan: 6-dodecanoyl-*N,N*-dimethyl-2-naphthylamine
MβCD: randomly methylated β-cyclodextrin
POPC: 1-palmitoyl-2-oleoyl-*sn*-glycero-3-phosphocholine
PY3174: (4-[2-(6-dibutylamino-5-fluoronaphthalen-2-yl)­vinyl]-1-(3-triethylammoniopropyl)­pyridinium dibromide
TMA-DPH: 4′-(trimethylammonio)­diphenylhexatriene
