# Supplementary material for: Disorder in Order-Related Membrane Biophysical Parameters: An In-Depth Analysis of Di-4-ANEPPDHQ Generalized Polarization
Source: J Phys Chem Lett. 2026 Apr 10;17(16):4723–31. doi: 10.1021/acs.jpclett.6c00048 (PMC13112443; doi:10.1021/acs.jpclett.6c00048)
Supplement: Supplementary file 1 [file jz6c00048_si_001.pdf]

Disorder in order-related membrane biophysical parameters: An in-depth analysis of di-4-ANEPPDHQ generalized polarization

Rosemary Chandrakanthi Kothalawala[a], Csenge Makay[a], Lajos Szente[b], Zoltan Varga[a], Gyorgy Panyi[a], Peter Nagy[a], Florina Zakany[a], Tamas Kovacs[a*]*

[a] Department of Biophysics and Cell Biology, Faculty of Medicine, University of Debrecen and MTA Centre of Excellence, Hungarian Academy of Sciences, Egyetem tér 1, Debrecen H-4032, Hungary

[b] CycloLab Cyclodextrin R&D Laboratory Ltd., Illatos u. 7., Budapest H-1097, Hungary

[*] florina.zakany@med.unideb.hu, kovacs.tamas@med.unideb.hu

Experimental Section

Cell Culture and Treatments

Chinese hamster ovary (CHO) cells obtained from the American Type Culture Collection (Manassas, VA) were grown according to their specifications. Cell were detached at a confluence of 80%–90% by trypsinization for spectrofluorometry, whereas the cells were grown on 8-well chambered coverglass (ibidi, Gräfelfing, Germany) for confocal microscopy. Before the measurements, cells were treated with 6-ketocholestanol (3β-hydroxy-5α-cholestan-6-one, 6KC) (Sigma Aldrich, St. Louis, MO), cholesterol (CHOL) (Sigma-Aldrich) or 7-dehydrocholesterol (7DHC) (Sigma-Aldrich) pre-complexed with methyl-beta-cyclodextrin (MβCD) (CycloLab Cyclodextrin R&D Laboratory, Budapest, Hungary) at sterol concentrations of 200 μM for 60 min at room temperature in normal Ringer's solution. Control samples were treated with the corresponding amounts of native MβCD.

Examination of Spectral Changes of Di-4-ANEPPDHQ Using Spectrofluorometry

Trypsinized control CHO cells and those treated with native or sterol-loaded MβCD complexes for 60 min were labeled with 5 μM di-4-ANEPPDHQ (Thermo Fisher Scientific, Waltham, MA) for 20 min at room temperature. After washing and resuspension at a concentration of 10^6/ml, fluorescence intensities were measured with a Fluorolog-3 spectrofluorometer (Horiba Jobin Yvon, Edison, NJ). The probe was excited at 488 nm and the emission spectrum was determined

between 500 nm and 750 nm with an increment of 1 nm using slits adjusted to 5 nm both on the excitation and emission sides. Generalized polarization (GP) of di-4-ANEPPDHQ fluorescence was calculated by integrating the measured intensities in two emission wavelength ranges between 500 and 570 nm (I_{blue}) and 660 and 735 nm (I_{red}) and according to the following formula:

$$GP = \frac{I_{blue} - I_{red}}{I_{blue} + I_{red}} \quad \text{(S1).}$$

Quantification of the Generalized Polarization of Di-4-ANEPPDHQ Using Confocal Microscopy

The GP of di-4-ANEPPDHQ in CHO cells, grown on 8-well chambered coverglass and treated and labeled as above, was quantified using an LSM880 confocal laser-scanning microscope (Carl Zeiss AG, Jena, Germany). Images were acquired at the midplane of cells using an excitation at 488 nm and detecting emitted fluorescence intensities in the wavelength ranges corresponding to I_{blue} and I_{red}, specified above. During quantitative image analysis performed in MATLAB (Mathworks, Natick, MA), images were segmented into membrane and nonmembrane pixels, which was followed by the identification of individual cells and calculation of the median value of di-4-ANEPPDHQ GP from the data of plasma membrane pixels for each individual cell after background subtraction using equation (1) as demonstrated in Figure 1C.

Molecular Dynamics Simulations

The structure of di-4-ANEPPDHQ was obtained from quantum mechanics (QM) optimized geometries and used for electrostatic potential (ESP) calculations. QM calculations were performed at the B3LYP/6-31G*//HF/6-31G* level of theory. The atomic partial charges were

derived with restrained electrostatic potential (RESP) methodology with a restraint weight of 0.01 from previous ESP calculations. GAFF force field parameters were used to describe the valence parameters. The di-4-ANEPPDHQ was embedded into a pre-equilibrated lipid bilayer composed of 1-palmitoyl-2-oleoyl-sn-glycero-3-phosphocholine (POPC) built with CHARMM-GUI at two different positions (Model 1 and Model 2 with the probe moved towards the center of the bilayer by 5 Å in the latter) with an initial tilt angle of approximately 30° relative to the membrane normal. The system was solvated in a TIP3P bilayer water shell and neutralized by adding chloride ions and, subsequently, 150 mM NaCl was added to simulate physiological salt concentration. The valence parameters of POPC were adopted from Lipid14.

Energy minimization was performed on the entire system for 1000 steps utilizing the steepest descent method and then 1000 steps employing the conjugate gradient method with a restrained of 100 kcal·mol^{-1}·Å^{-2} applied to the phospholipid head groups and dye heavy atoms. Then, the same optimization process was carried out, but with the restraint applied solely to the probe and the entire system was gradually heated to 300 K over 40 ps under the same restraint conditions. Subsequently, a 100-ps equilibration simulation was performed under the nPT ensemble at 300 K to balance the system density, using the CPU version of PMEMD from the AMBER22 software package with smaller restraints applied to the dye heavy atoms (10 kcal·mol^{-1}·Å^{-2}). All restraints were removed in 1-ns equilibration simulations to further optimize the system density, which were carried out with the GPU implementation of PMEMD. Finally, 250-ns production MD simulations were performed with generally stable fluorophore positions obtained within this time interval. Atomic coordinates of all atoms were recorded every 10 ps. The temperature and

pressure were controlled using the Langevin thermostat and the anisotropic Berendsen barostat, respectively. Bond lengths involving hydrogen atoms were constrained using the SHAKE algorithm, Van der Waals interactions were treated using a nonbonding cutoff of 10 Å, and electrostatic interactions were calculated with the particle mesh Ewald method.

The membrane thickness was measured based on the average distance between the P atoms in the upper and lower leaflets of bilayers. The tilt angle to membrane normal was determined based on N2 and N3 atoms, whereas the positional RMSD was calculated based on the heavy atoms of the dye. Distances of di-4-ANEPPDHQ to the interface were calculated between N2 and C23 atoms of di-4-ANEPPDHQ and the P atoms in phospholipids.

Statistical Analysis

Measured data are represented as mean ± SEM obtained from n independent samples containing approximately 100,000 cells for spectrofluorometry or n individual cells from five independent experiments for confocal microscopy, as indicated in figure legends. The p values were calculated by Tukey's HSD test carried out after significant differences were obtained for between-group effects in ANOVA. Differences were considered significant when $p < 0.05$ ($^{*}p < 0.05$, $^{**}p < 0.01$, $^{***}p < 0.001$, $^{****}p < 0.0001$).
